# Exploration of epithelial-mesenchymal transition-related lncRNA signature and drug sensitivity in breast cancer

**DOI:** 10.3389/fendo.2023.1154741

**Published:** 2023-07-19

**Authors:** Chengxin Li, Lewei Zheng, Gaoran Xu, Qianqian Yuan, Ziyang Di, Yalong Yang, Xingxing Dong, Jinxuan Hou, Gaosong Wu

**Affiliations:** ^1^ Department of Thyroid and Breast Surgery, Zhongnan Hospital of Wuhan University, Wuhan, China; ^2^ Department of Gastrointestinal Surgery and Department of Gastric and Colorectal Surgical Oncology, Zhongnan Hospital of Wuhan University, Wuhan, China

**Keywords:** epithelial-mesenchymal transition, breast cancer, long non-coding RNA, prognostic model, drug sensitivity

## Abstract

**Background:**

Breast cancer (BRCA) has become the most diagnosed cancer worldwide for female and seriously endanger female health. The epithelial-mesenchymal transition (EMT) process is associated with metastasis and drug resistance in BRCA patients. However, the prognostic value of EMT-related lncRNA in BRCA still needs to be revealed. The aim of this study is to construct an EMT-related lncRNA (ERL) signature with accuracy predictive ability for the prognosis of BRCA patients.

**Methods:**

RNA-seq expression data and Clinical characteristics obtained from the TCGA (The Cancer Genome Atlas) were used in the study. First, we identified the EMT-related lncRNA by the Pearson correlation analysis. An EMT-related lncRNAs prognostic risk signature was constructed using univariate Cox regression and Lasso-penalized Cox regression analyses. The model’s performance was validated using Kaplan-Meier (KM) survival analysis, ROC curve and C-index. Finally, a nomogram was constructed for clinical practice in evaluating the patients with BRCA and validated by calibration curve and decision curve analysis (DCA). We also evaluated the drug sensitivity of signature lncRNA and the tumor immune cell infiltration in breast cancer.

**Results:**

We constructed a 10-lncRNA risk score signature based on the lncRNAs associated with the EMT process. We could assign BRCA patients to the high- and low-risk group according to the median risk score. The prognostic risk signature showed excellent accuracy and demonstrated sufficient independence from other clinical characteristics. The immune cell infiltration analysis showed that the prognostic risk signature was related to the infiltration of the immune cell subtype. Drug sensitivity analysis proved ERLs signature could effectively predict the sensitivity of patients to common chemotherapy drugs in BRCA and provide guidance for chemotherapy drugs for high-risk and low-risk patients.

**Conclusion:**

Our ERL signature and nomogram have excellent prognostic value and could become reliable tools for clinical guidance.

## Introduction

Breast cancer (BRCA) has surpassed lung cancer to become the most diagnosed cancer worldwide, accounting for more than 30% of estimated new female cancer cases, and it is the second leading cause of female cancer-related death in the United States (1, 2). Even though various treatment strategies including surgical treatment, radiotherapy, chemotherapy and endocrine therapy, are applied for breast cancer, many challenge such as drug resistance to endocrine therapy and effective treatment for triple-negative breast cancer (TNBC) remain to be overcome (3, 4). In recent years, research aimed at immunotherapy has gradually increased. Although the conventional view is that breast cancer is a lesser immunogenic cancer type, the inhibitor anti-PD-1/PD-L1 inhibitor has become the predominant in breast cancer immunotherapy (5, 6). Understanding the molecular mechanism, tumor microenvironment (TME) as well as identifying effective therapeutic biomarkers for breast cancer are of great importance.

Epithelial-mesenchymal transition (EMT), a concept originating from developmental biology, refers to the epithelial cells transdifferentiating into motile mesenchymal cells (7). Recent research has shown

that EMT programs do not create mesenchymal cells; instead, they generate migrating cancer cells displaying both epithelial and mesenchymal characteristics (8). Based on the period and biological processes in which EMT occurs, these cells can be defined as three subtypes as follows: Type I EMT is associated with embryonic formation, Type II EMT occurs in wound healing and inflammation, and Type III EMT plays a significant role in tumors’ occurrence, development, and metastasis (9). In human cancer, EMT is usually an incomplete process. As the tumor progresses, neoplastic cells gradually transition from epithelial cells to quasi-mesenchymal cells with mesenchymal features, while complete mesenchymal cells generally do not appear (9). Previous research has demonstrated that the EMT process is related to breast cancer cell metastatic growth and resistance to chemotherapy (10, 11).

Long non-coding RNAs (lncRNAs) refer to a group of non-coding RNA consisting of more than 200 nucleotides (12). The effect of lncRNAs on the EMT process is mainly mediated through regulating pro-EMT transcription factors such as TWIST1/2, SNAIL, SLUG and ZEB1/2, which combine with the E-box of E-cadherin and suppress its expression to promote the EMT process (13). Research by Zhang et al. found that the long non-coding RNA LncATB acts as a sponge for the miRNA miR-200c and upregulates TWIST1 expression, inducing the EMT process associated with the poor prognosis in breast cancer (14). EMT is regulated by the lncRNA DLX-AS1/miR199b-5p/PXN axis to enhance TNBC cell proliferation and resistance to cisplatin (15). Therefore, identifying more lncRNAs that regulates the EMT process will have obvious benefit clinical therapy and aid in the understanding of molecular mechanism of tumor development.

In the present study, we constructed an EMT-related lncRNA signature prognostic model for BRCA patients and effectively distinguished the risk level of patients. Furthermore, we comprehensively evaluated the tumor immune microenvironment in BRCA patients and analyzed the correlation between EMT-related lncRNA signature and chemosensitivity.

## Materials and methods

### Data acquisition

We first downloaded the RNA-seq data of 1222 breast cancer samples (including 1109 breast cancer tumor tissue and 113 breast normal tissue) with the corresponding clinical characteristic information from the TCGA database. All sequencing data were summarized into the expression matrix of FPKM. The EMT-related gene set consists of 200 genes (**Supplementary Table 1**) obtained from the gene set “HALLMARK _EPITHELIAL_MESENCHYMAL_TRANSITON” in The Molecular Signatures Database. In order to ensure the quality of the study, patients’ follow-up time less than 30 days or with incomplete survival information were excluded. We randomly divided the remained 1039 BRCA patients into the training set (N=728) and the testing set (N=311) according to the ratio of 7:3. Training set and testing set patients’ clinical characteristics information was presented in **Supplementary Table 2**.

### Screening of EMT-related lncRNAs

We first acquired the annotation information of lncRNAs from the GENECODE (https://www.gencodegenes.org/human/) based on the RNA expression matrix annotated by Genome Reference Consortium Human Build 38 (GRCH38) and screened the lncRNAs using the *Perl* script. Pearson’s correlation analysis using the “cor.test” function in the R package “limma” was then performed between the expression matrix of EMT-related genes and lncRNAs (|correlation coefficient| >0.4 and *p* value<0.001).

### Construction and validation of the risk signature

Univariate Cox regression analysis was performed to explore overall survival-related prognostic ERLs in the training set, which were selected as candidate lncRNAs to construct the risk signature. We used the R package “glmnet” for LASSO-Cox regression analysis to prevent overfitting of the model and we constructed the risk signature by applying the coefficients obtained from multivariate Cox regression analysis. The calculation formula of risk score is as follows: risk score= $\sum_{i=1}^{n}\left(Coefi*Expi\right)$ where Expi stands for the relative expression values of lncRNA, Coefi stands for the lncRNA’s corresponding regression coefficient, testing set patients’ risk score was calculated as the same formula. Survival analysis was performed by the R package “survival” and “survimer”. We compared the survival difference between the high- and low-risk groups by the log-rank test. The time-dependent ROC curve was plotted to assess the prediction accuracy of the model for the survival status of breast cancer patients at 1-, 3-, and 5-years, and the R package “timeROC” was used for ROC analysis.

### Independent factor analysis and nomogram construction

Univariate and multivariate Cox regression analyses were performed to explore whether risk signature and corresponding clinical characteristics (age, T stage, N stage, M stage, ER status, PR status, and HER2 status) are independent prognostic factors in breast cancer patients. Using the R packages “rms” and “regplot”, a nomogram was constructed to predict and visualize the 1-, 3- and 5-year survival rates of breast cancer patients. The Harrell’s concordance index (C-index) could measure the proportion of pairs of patients for which the risk score assigned by the model is in concordance with the actual outcome (16). A calibration curve and the C-index were used to evaluate the accuracy of the nomogram. Decision Curve Analysis (DCA) is a statistical method used to evaluate the performance of a predictive model in a clinical decision-making context (17). We draw DCA decision curves to measure the clinical benefit of the nomogram, risk score signature, and other clinical characteristics.

### Principal component analysis

Principal component analysis is a widespread method for dimensionality reduction and identification of gene distribution patterns, and the R packages “scatterplot3d” were performed to visualize the PCA diagram.

### Multivariate ROC curve

The multivariate ROC curve evaluated the predictive accuracy of the risk score and various clinical characteristics on the patient’s survival status, and further assessed the predictive accuracy of each factor by the C-index.

### Prediction of chemotherapeutic sensitivity

We downloaded chemotherapy drug response data from the Genomics of Drug Sensitivity in Cancer (GDSC) database. The R package “pRRophetic” was utilized to predict chemotherapeutic sensitivity to the chemotherapy and targeted drugs recommended in the NCCN guidelines in BRCA patients. The Half-maximal inhibitory concentration (IC50 value), representing the concentration of drug required to inhibit 50% of cell growth, is a valid indicator to assess the sensitivity of breast cancer cells to chemotherapeutic drugs. IC50 differences between high- and low-risk groups were compared using the Wilcoxon test, and p<0.05 was considered statistically significant.

### Gene set enrichment analysis

We applied Gene Set Enrichment Analysis v 4.1 software for signature lncRNAs pathway enrichment analysis for high- and low-risk groups of BRCA patients. c2.cp.kegg.v7.4.symbol.gmt was downloaded as the reference file, and adjusted p values< 0.05 and FDR< 0.25 were defined as statistically significant.

### Evaluation of the tumor immune microenvironment

We used the CIBERSORT algorithm (https://cibersort.stanford.edu/) to calculate the relative abundance of 22 immune cells, including B cells, CD4+ T cells, CD8+ T cells, macrophages, dendritic cells, plasma cells, NK cells, mast cells, eosinophils, neutrophils and monocytes in each sample. Only samples with CIBERSORT p<0.05 were retained for further analysis. The infiltration of immune cells and expression of immune checkpoint genes in patients belonging to high- and low-risk groups were analyzed using the Wilcoxon test. The Spearman correlation analysis determined the relationship between immune cell infiltration and the signature risk score. The Tumor Immune Dysfunction and Exclusion (TIDE) algorithm was utilized to predict each patient’s response to immunotherapy.

### Cell culture and RT-qPCR analysis

The MDA-MB-231 and MCF-7 human breast cancer cell lines and the MCF-10A normal breast epithelial cell line were obtained from Procell (Wuhan, China). The MCF-10A cell line was cultured in MCF-10A specialized medium CM-0525 (Procell, Wuhan, China). The other cell-lines were cultured in DMEM (Irvine Scientific, Carlsbad, CA, USA) supplemented with 10% foetal bovine serum (FBS). RNA was extracted from cells using the TRIzol method with RNA isolater total RNA extraction reagent (Vazyme, China). A HiScript II QRT SuperMix (Vazyme, China) kit was used to reverse transcription. qRT-PCR was conducted using the 2X Universal SYBR Green Fast qPCR Mix (Abclonal, China) kit and the CFX96 Real-time PCR Detection System (Bio-Rad, USA). We searched the sequences of lncRNAs in the LNCipedia (https://lncipedia.org). Unfortunately, we could not find the lncRNA sequences for AC036108.3 and AL390755. Thus, we selected other 8 lncRNAs for *in vitro* validation. The primers were synthesized by the Tsingke Biotechnology Limited Company (Wuhan, Hubei, China). The GAPDH was selected as the internal housekeeping gene, and the relative gene expression was calculated by the 2^-ΔΔCt^ method. Each qRT-PCR assay was repeated three times. We also compared the expression of signature lncRNAs in the subtypes of hormone receptor positive, HER2-amplified and triple-negative breast cancer in the TCGA dataset.

### Induction and verification of EMT

To induce EMT in breast cancer cells, we treated breast cancer MCF7 cells with lactic acid. Lactic acid was purchased from ALADDIN (L118493-1g, Shanghai, China). Lactic acid was diluted to concentrations of 5 mM and 10 mM with medium and treated MCF7 cells for 24 hours. The expression levels of EMT markers E-cadherin and N-cadherin were detected by Western blot. N-cadherin (22018-1-AP, 1:2000), E-cadherin (20874-1-AP, 1:5000) and GAPDH (60004-1-Ig, 1:5000) antibodies were purchased from Proteintech (Wuhan, China). The Bio-Rad ChemiDoc^®^ Touch Imaging System (Bio-Rad) was applied for the visualization of protein bands.

### Statistical analysis

The statistical analysis was conducted by R (version 4.1.2) and Perl (version 5.24) software. A random sequence was generated by SPSS 22.0. Survival curves were evaluated by Kaplan-Meier analysis and log-rank test. p value< 0.05 was considered significantly significant. The Wilcoxon test was used to assess significant differences between the two groups. We plotted ROC curves to evaluate the prediction accuracy of the prognostic signature with area under the ROC curve (AUC) values of 0.6-0.7, 0.7-0.9 and 0.9-1.0 were defined as acceptable, moderate and high accuracy, respectively.

## Results

**Figure 1:**
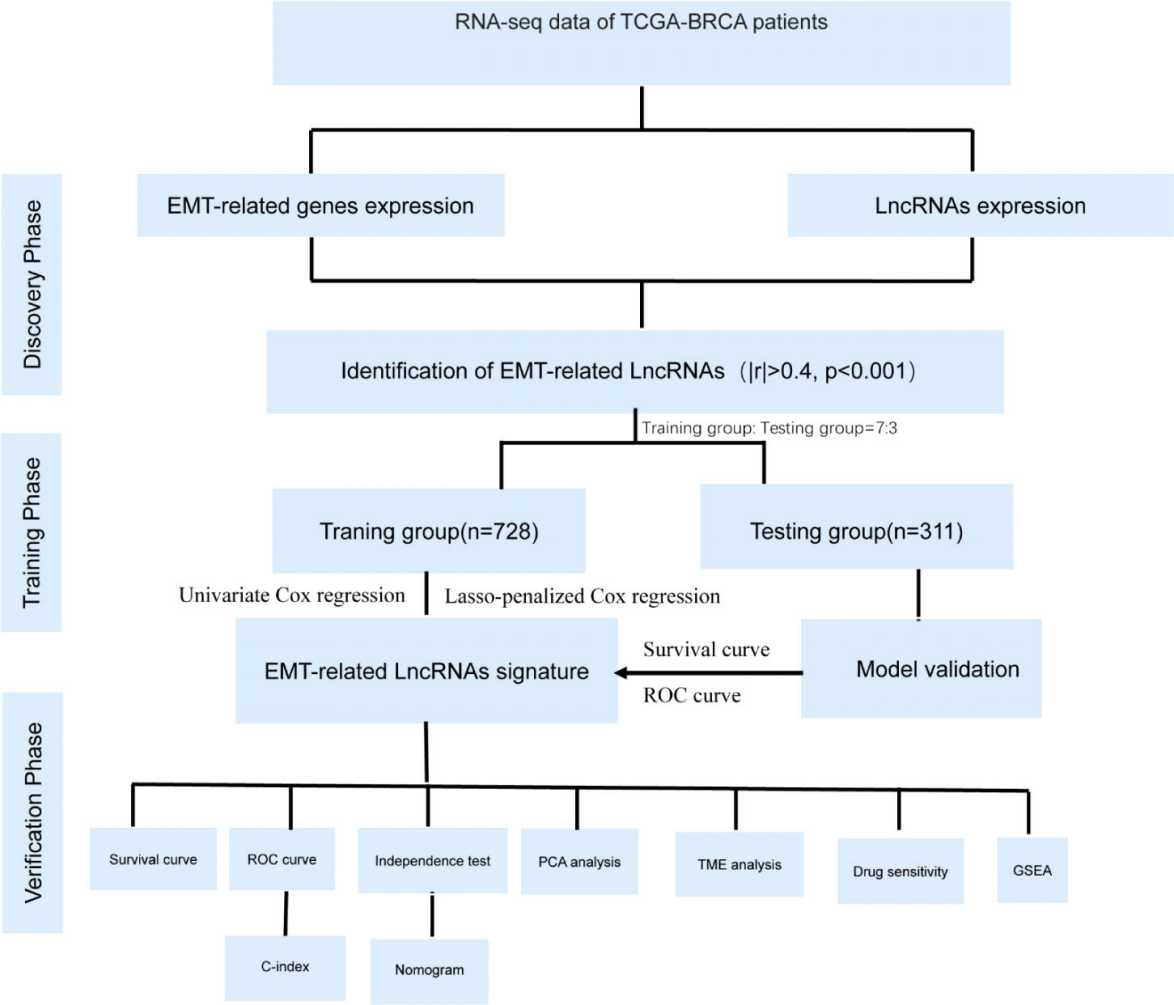
The flow chart of this study: flow chart showing flow identification of EMT- related lncRNAs, development and validation of novel 10-lncRNA based risk score signature.

To increase the readability of our research for readers, we presented a research flow chart in **Figure 1**.

### Identification of ERLs associated with prognosis

We first used the GENECODE to annotate RNA-seq data in the TCGA-BRCA dataset, and a total of 13162 lncRNAs were annotated. Pearson’s correlation analysis identified 469 ERLs with |correlation coefficient| >0.4 and *p* value<0.001. Moreover, 32 ERLs associated with BRCA patient prognosis were obtained by univariate Cox regression analysis in the training set (p<0.05). 21 were identified as prognostic good factors (hazard ratio<1), and 11 lncRNAs were identified as bad prognostic factors in BRCA patients (hazard ratio>1). These prognosis-related ERLs are listed in **Supplementary Table 3** and shown in the forest plot (**Supplementary Figure 1**).

### Construction of a 10-ERL prognostic signature of breast cancer

**Figure 2:**
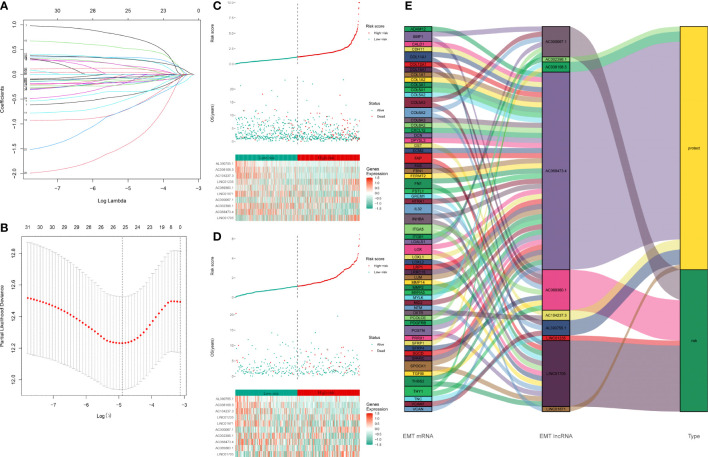
Construction of EMT-related lncRNAs prognostic signature: **(A, B)** LASSO coefficients calculated by the LASSO analysis **(C)** Distribution of training set patient’s risk score and outcomes. The heatmap of 10 signature lncRNAs expression profiles in the training set. **(D)** Distribution of testing set patient’s risk score and outcome. The heatmap of 10 signature lncRNAs expression profiles in the testing set. **(E)** The Sankey plot of the EMT-related lncRNA signature. The type “risk” indicates that the high expression of lncRNA is a risk factor for the prognosis. The type “protect” indicates that the high expression of lncRNA is a protective factor for the prognosis. The line between EMT mRNA and EMT lncRNA indicates they might have a potential regulation relationship.

Based on 32 prognosis-related ERLs, we performed LASSO regression analysis to prevent model overfitting and selected 25 optimal ERLs as candidates for signature construction (**Figures 2A, B**). Furthermore, an ERL signature risk score model for breast cancer patients was constructed using the 10 independently associated prognostic ERLs screened by multivariate Cox regression analysis (**Supplementary Table 4**). The risk score was calculated as the following formula: risk score=-0.678*AC002398.1-0.493*AC036108.3 + 0.311*LINC01705 + 1.280*AC069360.1-1.355*AL390755.1 + 0.818*AC000067.1-0.256*LINC01871 + 0.396*LINC01235-2.335*AC068473.4-1.635*AC104237.3. According to the cut-off point risk score in the training set, patients in the training set (**Figure 2C**) and testing set (**Figure 2D**) were divided into high- and low-risk groups. As the risk score increased, more death events occurred in BRCA patients. The heatmaps show the correlation between risk status and expression of signature lncRNAs. The risk lncRNAs were highly expressed in the high-risk group, while the protective lncRNAs were highly expressed in the low-risk group. The Sankey plot in **Figure 2E** shows the relationship between EMT-related mRNAs and EMT-related lncRNAs.

### Evaluation of the prognostic value of EMT-related lncRNAs in breast cancer

**Figure 3:**
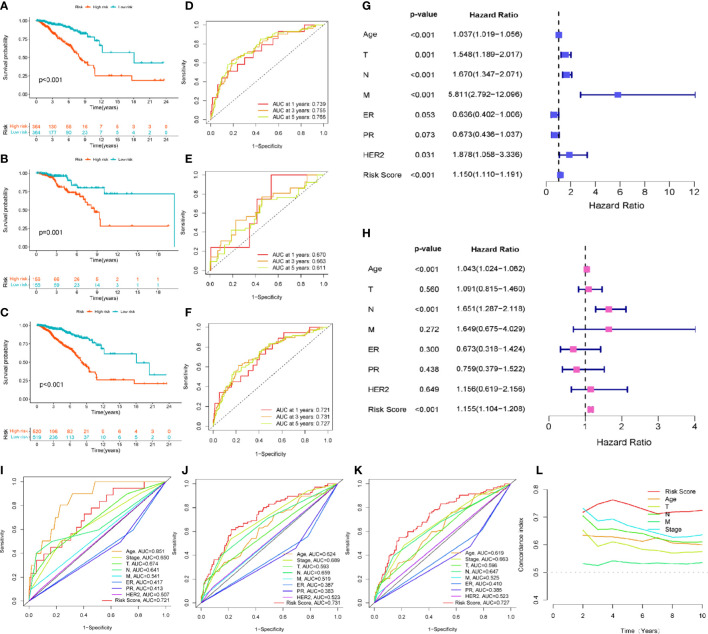
Evaluation and validation of the EMT-related lncRNAs prognostic signature’s prognostic value: **(A)** Kaplan-Meier survival curve analysis for the patients in training set. **(B)** Kaplan-Meier survival curve analysis for the patients in testing set. **(C)** Kaplan-Meier survival curve analysis in all patients set. **(D)** The 1-, 3-, 5-year ROC curve of overall survival for the patients in training set. **(E)** The 1-, 3-, 5-year ROC curve of overall survival for the patients in testing set. **(F)** The 1-, 3-, 5-year ROC curve of overall survival in all patients set. **(G, H)** Univariate and multivariate Cox regression analysis for signature risk score and clinical characteristics. **(I)** The multivariate ROC analysis for 1-year overall survival in all patients set **(J)** The multivariate ROC analysis for 3-year overall survival in all patients set **(K)** The multivariate ROC analysis for 3-year overall survival in all patients set **(L)** C-index analysis for the signature risk score and clinical characteristics.

Kaplan-Meier survival analysis with a log-rank test was performed to evaluate the potential value of EMT-related lncRNAs in predicting the prognosis of BRCA patients. As the survival curves show in **Figures 3A–C**, the prognosis of high-risk BRCA patients was significantly worse than that of low-risk BRCA patients in the training set (p<0.001), testing set (p=0.001) and all patients set (p<0.001). We next plotted the ROC curve to validate the predictive accuracy of the ERL signature in the training and testing sets. The area under the ROC curve (AUC) values in the training set (**Figure 3D**) were 0.739 (1 year), and 0.755 (3 years), AUC values in the testing set were 0.766 (5 years) and 0.670 (1 year), 0.663 (3 years), and 0.611 (5 years) (**Figure 3E**), and AUC values in the all patients set were 0.721 (1 year), 0.731 (3 years), 0.727 (5 years) (**Figure 3F**). After combining multiple clinical characteristics with the ERL signature for univariate Cox regression analysis and multivariate Cox regression analysis, the ERL risk score was identified as an independent prognostic risk factor in BRCA patients (p<0.001, Hazard Ratio=1.155 95%CI=1.104- 1.208). In addition, age and lymph node metastasis were identified also independent prognostic risk factors for BRCA patients (age: p<0.001, Hazard Ratio=1.043 95%CI=1.024-1.062; lymph node metastasis: p<0.001 Hazard Ratio=1.651 95%CI=1.287-2.118) (**Figures 3G–H**). Multivariate ROC curve analysis was performed to compare our ERL signature to the TNM staging system and other clinical characteristics. The ERL risk score had higher accuracy in predicting OS in BRCA patients at 3 and 5 years than the other clinical characteristics, but age had a higher predictive accuracy in 1-year OS prediction (**Figures 3I–K**). The concordance index (C-index) is also an effective tool to evaluate the accuracy of clinical factors in predicting patient prognosis. According to the C-index, the ERL risk score was significantly more accurate than the other clinical factors in predicting the prognosis of BRCA patients (**Figure 3L**). The above results demonstrated that the EMT-related lncRNA signature can effectively predicts the OS of patients with high accuracy.

### Construction and validation of the nomogram

**Figure 4:**
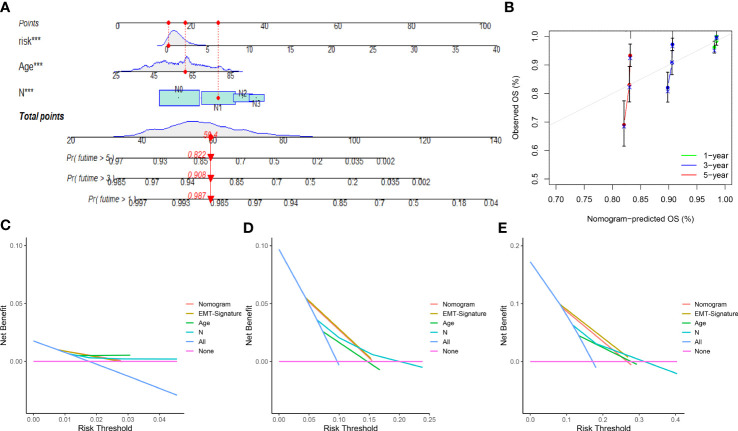
Nomogram and calibration curve of prognostic risk model: **(A)** Nomogram based on EMT-related lncRNAs signature and clinical characteristic to predict 1-, 3- and 5-year overall survival of breast cancer patients. **(B)** The calibration curves for 1-, 3- and 5-year nomogram. **(C)** Decision curves for 1-year overall survival. **(D)** Decision curves for 3-year overall survival. **(E)** Decision curves for 5-year overall survival. ***p<0.001.

We next constructed a nomogram based on the independent factors (ERL risk score, age and lymph node metastasis) obtained from multivariate Cox regression analysis to quantify the predictors of 1-, 3-, and 5-year prognosis in breast cancer patients (**Figure 4A**). The accuracy of the nomogram was validated by calibration curves and C-index, the C-index was 0.749 (95%CI=0.700-0.798), which showed that the nomogram had moderate predictive accuracy for BRCA patient outcomes at 3 and 5 years (**Figure 4B**). Decision curve analysis (DCA) was used to analyze the benefit of each model and clinical characteristics to the patient for selecting the optimal model. The difference in the 1-year decision curves was not apparent (**Figure 4C**), but both the nomogram and EMT-related lncRNA signatures showed higher predictive accuracy than the other clinical characteristics in the 3- and 5-year decision curves (**Figures 4D, E**). The above results indicated that the nomogram and EMT-related lncRNA signature have a better prediction ability for the 3-year and 5-year survival rates of patients compared to other clinical characteristics but that the 1-year survival rate of patients is more affected more by the age of the patients.

### Applicability of the ERL signature in various clinical subgroups

To further evaluate the utility of the ERL signature for patients with different clinical characteristics (age, clinical stage, T stage, N stage, M stage, ER receptor status, PR receptor status, and HER2 receptor status), subgroup survival analysis was performed. Except for patients without distant metastasis and HER2 receptor positivity, the ERL signature effectively determined the prognostic risk of patients in the clinical characteristic subgroups (p<0.05) (**Supplementary Figure 2**).

### The ERL signature is associated with chemosensitivity

**Figure 5:**
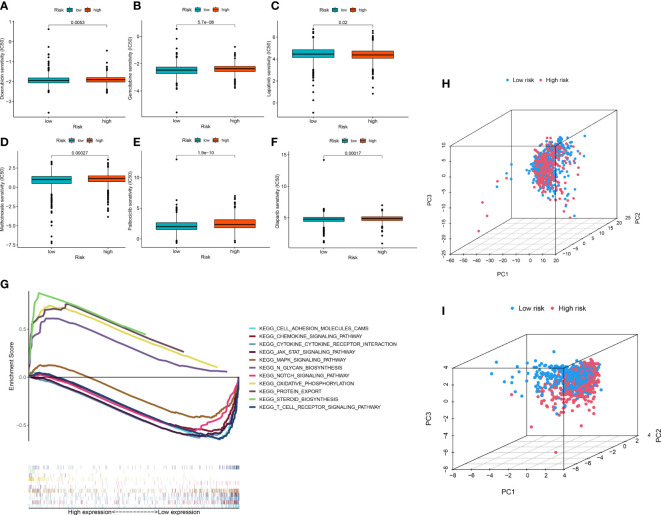
**(A–F)** Drug sensitivity analysis **(G)** Signaling pathway enrichment analysis between high- and low-risk group patients via GSEA. **(H, I)** PCA of breast cancer patients’ distribution analysis.

To improve the application value of the ERL signature for chemotherapy in BRCA patients, we assessed the relationship between the sensitivity of commonly used breast cancer chemotherapy drugs and risk signatures. The results showed that the resistance to doxorubicin (**Figure 5A**), gemcitabine (**Figure 5B**), methotrexate (**Figure 5D**), palbociclib (**Figure 5E**), olaparib (**Figure 5F**) in the high-risk group was higher than that in the low-risk group, while patients in the high-risk group were less resistant to lapatinib (**Figure 5C**) than those in the low-risk group. These results demonstrated that the ERL signature can effectively predicts the sensitivity of patients to common chemotherapy drugs in BRCA, thereby providing guidance for chemotherapy drugs for high-risk and low-risk patients.

### Signaling pathway enrichment based on the ERLs signature

GSEA was performed to identify essential signaling pathways significantly associated with BRCA samples from the high-risk group and BRCA samples from the low-risk group. BRCA samples from the high-risk group were significantly enriched in steroid biosynthesis, oxidative phosphorylation, protein export, and N glycan biosynthesis. In contrast, samples from the low-risk group were significantly enriched in the chemokine signaling pathway, JAK-STAT signaling pathway, cytokine receptor interaction, T cell receptor signaling pathway, cell adhesion molecules, Notch signaling pathway and MAPK signaling pathway (**Figure 5G**).

### Association between ERLs and patient risk distribution

To visualize the distribution pattern of patients in the high- and low-risk groups, we performed PCA analysis using ERL expression levels. The results showed that the PCA using all ERLs had a weak ability to distinguish patients in the high- and low-risk groups (**Figure 5H**), while the PCA analysis using ERLs in the risk model significantly distinguished patients into two clusters (**Figure 5I**), which further validated the predictive effect of the model on patient prognosis.

### ERLs are associated with the tumor immune microenvironment

**Figure 6:**
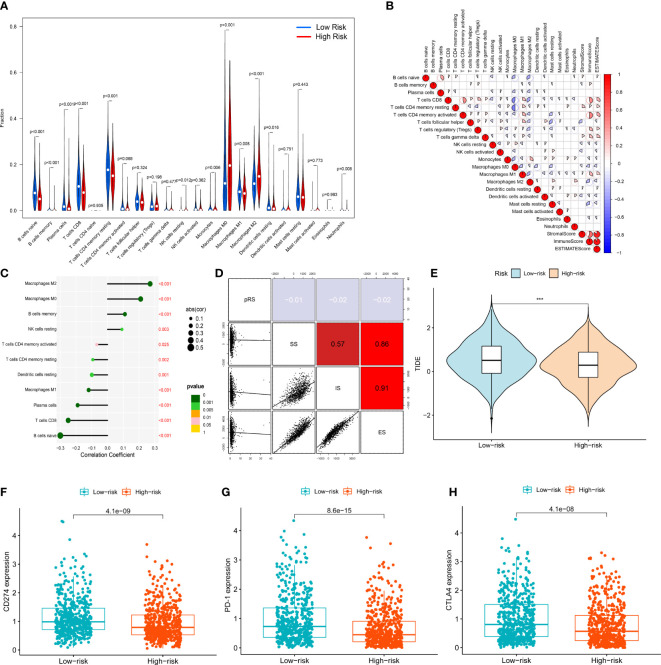
Immune cell infiltration analysis: **(A)** Vioplot for the immune cell abundances differences between high- and low-risk group. **(B)** The correlation between immune cells and immune infiltration-related score (stromal score, immune score, ESTIMATE score) **(C)** Lollipop plot showed the correlation between immune cells and signature risk score. **(D)** The correlation between signature risk score and immune infiltration-related score (stromal score, immune score, ESTIMATE score). **(E)** Immune therapy response difference analysis for high- and low-risk group BRCA patients. **(F)** The correlation between expression of CD274 and risk. **(G)** The correlation between expression of PD-1 and risk. **(H)** The correlation between expression of CTLA4 and risk. ***p<0.001.

Based on the CIBERSORT algorithm, we measured the infiltration of 21 TIICs (excluding CD4 naive T cells) in entire set of patients and further compared them between patients in the high- and low-risk groups (**Figure 6A**). In the high-risk group, the infiltration levels of Macrophages M0, Macrophages M2, B cells memory, NK cells resting were significantly increased (p<0.05), while the infiltration levels of Macrophages M1, B cells naive, Plasma cells, T cells CD8, T cells CD4 resting, Infiltration levels of Monocytes, Dendritic cells resting, Neutrophils were significantly reduced (p<0.05). We further assessed the correlation between immune cells and immune/stromal/ESTIMATE scores (**Figure 6B**). The results of the Spearman correlation test between the risk score and the level of immune cell infiltration, the results were visualized using a lollipop plot (**Figure 6C**). The results indicated that Macrophages M0, Macrophages M2, B cells memory, and NK cells resting were significantly associated with higher risk scores, and they also demonstrated that Macrophages M1, B cells naive, plasma cells, T cells CD8, T cells CD4 resting, T cells CD4 activated and dendritic cells resting were significantly correlated with lower risk scores (p<0.05). The correlation between the risk score and immune/stromal/ESTIMATE score was weak (**Figure 6D**). To identify patient populations with potential benefits from immunotherapy, TCGA-BRCA immunotherapy responses were analyzed based on the TIDE algorithm, in which the efficacy of immune checkpoint blockade (ICB) in patients with high TIDE scores is poor. The results showed that the efficacy of immunotherapy in the high-risk group was significantly better than that in the low-risk group. Therefore, these results suggested that patients in the high-risk group should receive immunotherapy (**Figure 6E**). In addition, the expression of the well-known immune checkpoint genes, namely, PD-L1, PD-1 and CTLA4 was lower in the high-risk group compared to the low-risk group, which was consistent with the TIDE score (**Figures 6F–H**).

### Expression of EMT signature lncRNAs *in vitro* validation by RT-qPCR analysis

**Figure 7:**
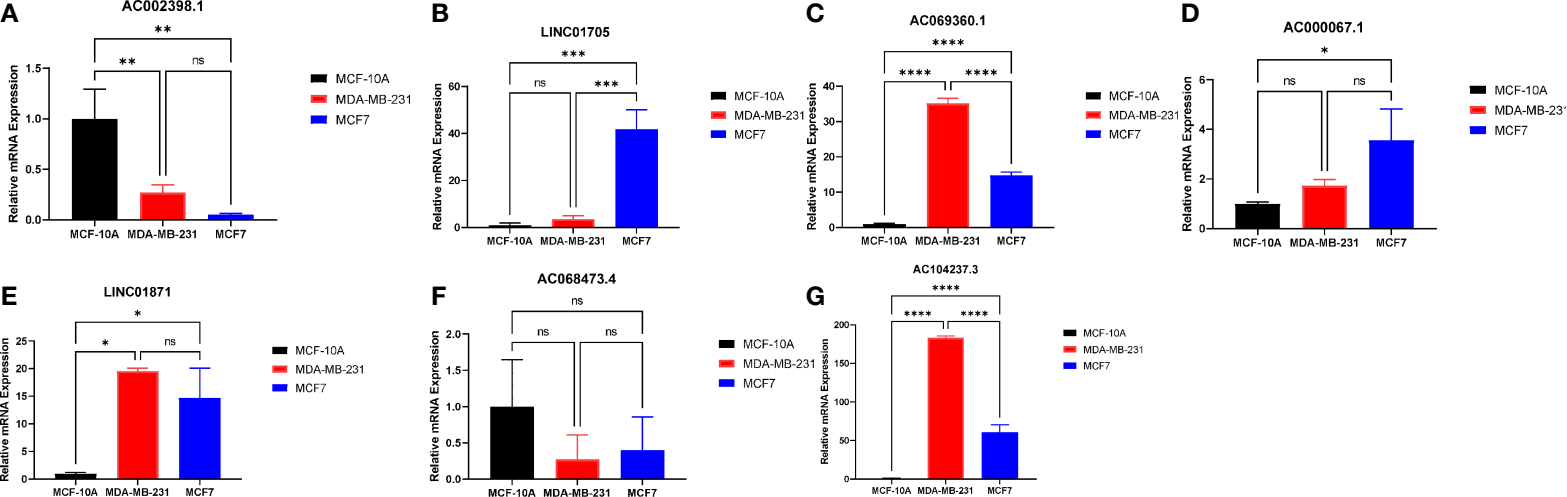
RT-qPCR analysis for the EMT signature lncRNAs: **(A)** AC002398.1 **(B)** LINC01705 **(C)** AC069360.1 **(D)** AC0000671 **(E)** LINC01871 **(F)** AC068473.4 **(G)** AC104237.3; (ns, not significant, * p<0.05, ** p<0.01, *** p<0.001, **** p<0.0001).

The sequences of lncRNAs were presented in the **Supplementary Table 5**. Compared to breast cancer cell lines, the expression of *AC002398.1* was higher in breast epithelial cell lines (**Figure 7A**). The expression of *AC069360.1*, *LINC01871* and *AC014237.3* was higher in the breast cancer cell lines compared to the breast epithelial cell lines (**Figures 7C, D, F, H**). *LINC01705* and *AC000067.1* were only significantly highly expressed in the MCF-7 cell line (**Figures 7B, E**). The mean expression level of *AC068473.4* was lower in the breast cancer cell lines than in the normal breast epithelial cell line but did not attain statistical significance (**Figure 7G**). The ct-value of LINC01235 was over 45, in the MCF-10A cell line, we did not include it in our data. The results of expression levels of the signature lncRNAs in different subtypes of breast cancer are shown in the **Supplementary Figure 3**.

### *In vitro* experiments confirm that EMT lncRNA is related to EMT

**Figure 8:**
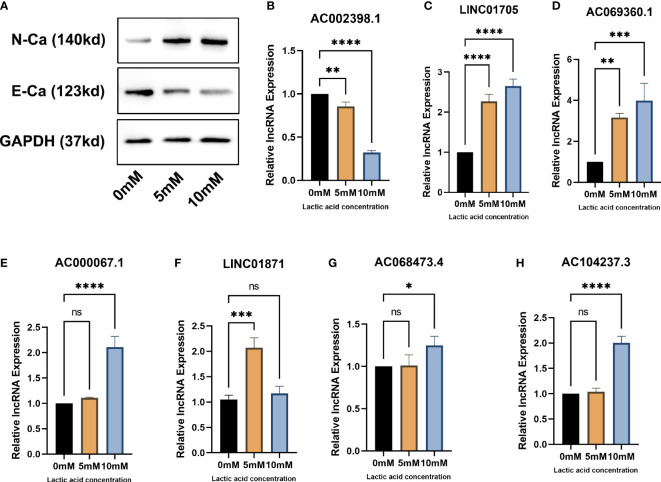
*In vitro* experiments verify that EMT-related lncRNAs are associated with EMT: **(A)** Western blot proves that lactic acid induces EMT in breast cancer cells **(B–H)** RT-qPCR analysis confirmed that there were differences in the expression of EMT-related lcnRNAs in different EMT status of breast cancer cells. * p<0.05, ** p<0.01, *** p<0.001, **** p<0.0001.

After lactic acid treatment, the expression of N-cadherin was significantly increased while the expression of E-cadherin was significantly decreased in MCF-7 cells, suggesting that lactic acid significantly induced EMT in breast cancer cell lines (**Figure 8A**). In subsequent RT-qPCR **(** **Figures 8B–H**) analysis, we found that the expression of EMT-related lncRNAs such as AC069360.1, LINC01871, AC014237.3, LINC01705, AC000067.1 and AC068473.4 increased to varying degrees after lactic acid treatment. It shows that this part of lncRNAs may be related to the promotion of EMT in breast cancer. The expression of AC002398.1 decreased after lactic acid treatment, reflecting that it can inhibit the EMT of breast cancer cells.

## Discussion

Breast cancer is a malignancy with high incidence and disease burden for females globally, and the identification of novel biomarkers for prognosis and treatment is urgently needed (18). The epithelial-mesenchymal transition process plays a vital role in tumor metastasis was first revealed in breast cancer. This essential contribution triggered numerous studies on the correlation between EMT and tumors (19). During EMT, epithelial cells lose epithelial features and gain migratory abilities of mesenchymal cell traits promoting tumor progression (20). EMT-inducing chemotherapy resistance in breast cancer has been increasingly gaining attention. Wnt3 overexpression activates the Wnt/β-catenin signaling pathway inducing the EMT process, which affords HER2-positive breast cancer cells obtain trastuzumab-resistant characteristics (21). Tamoxifen is the first-line treatment for estrogen receptor (ER)-positive breast cancer, but drug resistance appears in 30% of ER-positive BRCA patients and accounts for nearly all distant metastases. Yin et al. found that Cx43 deficiency increases EMT-mediated tamoxifen resistance through the c-Src/PI3K/Akt pathway (22). These studies indicate that the inhibition of EMT can significantly prevent drug resistance and improve the prognosis. Thus, the study of EMT-related biomarkers may provide a novel predictor for prognosis and effective therapy for breast cancer patients. Previous studies have mainly focused on the EMT-related genes in breast cancer (23, 24). Due to the essential role that lncRNAs play in gene transcription and posttranscriptional regulation, we aimed to identify EMT-related lncRNAs in breast cancer and explore the relationship among prognosis, the tumor immune microenvironment and chemotherapy sensitivity.

In the present study, we constructed a prognostic signature based on the 10 EMT-related lncRNAs. Among these ten lncRNAs, LINC01705, LINC01235, LINC01871 and AL390755.1 have been correlated with breast cancer progression in previous studies. Li et al. found that LINC01705 can serve as a competitive endogenous RNA for sponging miRNA miR-186-5p, regulating TPR expression and further influencing the BRCA progression (23). The LINC01235-mediated EMT process promoting tumor metastasis has been confirmed in gastric cancer (25, 26). LINC01235 is associated with poor disease-free survival (DFS) and OS for BRCA patients according to Kaplan-Meier survival analysis (27). Moreover, LINC01871 and AL390755.1 are involved in other breast cancer lncRNA prognostic signatures (28). As shown by the ROC curve analysis, the ERL signature showed excellent predictive accuracy. To benefit clinical practice guidance, we also constructed a nomogram to predict 1-, 3-, and 5-year OS for BRCA patients. The TNM staging system and molecular typing are widely used tools for predicting BRCA patient prognosis. Multivariate ROC analysis and DCA indicated that our ERL prognostic signature and nomogram had better prediction ability than the TNM staging system, but more clinical evidence is required for validation.

The CIBERSORT algorithm was used to assess the relationship between immune cell infiltration and ERLs in the present study. A substantial number of studies have demonstrated that the EMT process is strongly impacted by the tumor immune microenvironment, further promoting the invasion and migration of the tumor cells (29–31). The research and regulation of immune cell infiltration characteristics in tumor tissue is an important basis for current clinical research to select appropriate immunotherapy patient types. Some studies have found that local tumor tissues with a good prognosis show significant infiltration of CD8+ T cells, Th1 cells, NK cells, DC1 cells and M1 macrophages, and such immune cell populations show strong tumor-killing effects in the local microenvironment. Poor prognosis and metastatic tumors show obvious immune tolerance/immune escape features, including an increased proportion of M2 macrophages and DC2 cells and increased Th2 cells as well as high production of IL-10 and TGF-β among other characteristics of Treg cells (32). In our research, the high-risk group of BRCA patients had higher M2 macrophage infiltration levels than the low-risk group. Santarpia et al. revealed that the expression of EMT-related tyrosine kinase AXL is positively associated with the infiltration levels of M2 macrophages, especially in the TNBC cells with mesenchymal features. This phenomenon showed that the mesenchymal-like cells support the crosstalk with tumor-associated macrophages (TAMs), and targeting AXL inhibits the invasion of TNBC by suppressing the EMT process and TAMs (33). Zhang et al. identified a risky positive feedback loop between TAMs and breast cancer cells; TGF-β1 released by TAMs induces the EMT process via the PI3K-Akt pathway in the breast cancer cells, and breast cancer cells also release TGF-β1 to maintain the TAM-like phenotype for macrophages (34). The expression of PD-L1 is promoted by EMT in carcinoma cells, and tumors that express higher EMT scores have been found to respond better to antibodies targeting CTL-A4, PD1, and PD-L1, as well as express increased levels of other immune checkpoint markers (35). The above studies provide possible explanations for the immune infiltration level results in the present study. The ERLs signature low-risk group of BRCA patients had higher TIDE scores and immune checkpoint gene expression, indicating that these patients may benefit more from the immune therapy.

Many previous studies have suggested that inhibiting the EMT process in tumor cells may be a method for chemotherapy resistance in breast cancer. Doxorubicin, an anthracycline drug, is widely used in neoadjuvant chemotherapy for BRCA patients, but ERα-negative BRCA patients are less sensitive to doxorubicin than ERα-positive BRCA patients. Ding et al. found that doxorubicin treatment enhances the expression of the crucial EMT transcription factors Snail and Twist via the ERα signal pathway, which promotes the EMT process, resulting in the doxorubicin resistance in the ERα-positive BRCA cells (36). MicroRNA miR-137 and G-protein coupled receptor 30 (GPR30) have also been confirmed to have a target effect on doxorubicin resistance by regulating the EMT process (37, 38). Moreover, NLRP3 enhances gemcitabine resistance in TNBC cells through the EMT/IL-1β/Wnt/β-catenin signaling pathway (39). The researches about lapatinib resistance in relation to EMT showed that laptinib-induced MET could significantly suppress EMT process (40, 41). Based on the significant effect of lapatinib in suppressing EMT, we speculate that it is more appropriate for use in high-risk patients. According to the results of the chemotherapy sensitivity analysis based on the ERL signature, BRCA patients in the low-risk group were more suitable to be treated with doxorubicin, gemcitabine, methotrexate, palbociclib, and olaparib, while lapatinib was more appropriate for treating the BRCA patients in the high-risk group BRCA patients.

Through a series of experiments, we proved that AC069360.1, LINC01871, AC014237.3, LINC01705, AC000067.1 and AC068473.4 may be related to the promotion of EMT in breast cancer cells, while AC002398.1 may be related to the inhibition of EMT in breast cancer cells. This is a development first reported by us.

However, some limitations in our study should be mentioned: Our research was mainly based on the public database with bioinformatics methods, and whether EMT-related lncRNA effect on the EMT process should be further validated *in vitro* and *in vivo* experiments. The external validation of the EMT-related lncRNAs signature is lacking in the current study due to the lack of complete lncRNA expression data in the external dataset, such as the GEO and METABRIC. Our ERL signature and nomogram have no examples yet for clinical application, we hope that it will be clinically applicable in the near future. Furthermore, the median follow-up period was less than 3 years, but our future studies will involve longer median follow-up periods to validate these results.

In conclusion, the present study constructed an EMT-related lncRNA signature that predicts the prognosis of BRCA patients, and it also revealed the immune microenvironment and drug sensitivity, providing new insight for breast cancer clinical precision therapy.

## Data availability statement

The original contributions presented in the study are included in the article/**Supplementary Material**, further inquiries can be directed to the corresponding authors.

## Author contributions

CL, JH and GW designed the work, GX and LZ completed *in vitro* experiments, CL, GX and LZ performed the data analysis. CL, LZ and ZD plotted the figures. CL and LZ wrote the manuscript. YY, QY and XD reviewed and revised the manuscript. All the authors have read and approved the manuscript.
